# Supplementary material for: DNA barcoding unveils a high diversity of caddisflies (Trichoptera) in the Mount Halimun Salak National Park (West Java; Indonesia)
Source: PeerJ. 2022 Dec 12;10:e14182. doi: 10.7717/peerj.14182 (PMC9753737; doi:10.7717/peerj.14182)
Supplement: Supplemental Information 1 [file peerj-10-14182-s001.docx]

| No. | Associated taxa | larva | adult |
| --- | --- | --- | --- |
| I | *Diplectrona gombak* | Hydropsychidae (13.4)\| INDOBIOSYS-CCDB24595-E06 | *Diplectrona gombak*\|INDOBIOSYS-CCDB30314-C06, n=3 |
|  |  | Hydropsychidae (6.20)\| INDOBIOSYS-CCDB24595-C03 |  |
|  |  | Hydropsychidae (6.13)\| INDOBIOSYS-CCDB26127-H05 |  |
| II | *Diplectrona pseudofasciata* | Hydropsychidae (10.14)\| INDOBIOSYS-CCDB25378-E04-E08 | *Diplectrona pseudofasciata*\|INDOBIOSYS-CCDB30314-B03 |
|  |  | Hydropsychidae (12.1)\| INDOBIOSYS-CCDB24595-E01 |  |
| III | *Hydropsyche saranganica* | Hydropsychidae (4.8)\| INDOBIOSYS-CCDB24595-A07 | *Hydropsyche saranganica*\|INDOBIOSYS-CCDB26180-B12 |
|  |  | Hydropsychidae (6.1)\| INDOBIOSYS-CCDB24595-B11 | *Hydropsyche saranganica*\|INDOBIOSYS-CCDB26180-C02 |
| IV | *Cheumatopsyche globosa/lucida-*complex | Hydropsychidae (3.1)\| INDOBIOSYS-CCDB24595-A02 | *Cheumatopsyche globosa*\|INDOBIOSYS-CCDB30314-G01  *Cheumatopsyche lucida*\|INDOBIOSYS-CCDB26178-F03  *Cheumatopsyche* sp.\|INDOBIOSYS-CCDB26178-F02  *Cheumatopsyche* sp.\|INDOBIOSYS-CCDB30314-G09 |
| V | *Hydromanicus flavoguttatus* | Hydropsychidae (20.1)\|INDOBIOSYS-CCDB24595-H07 | *Hydromanicus flavoguttatus* \|INDOBIOSYS-CCDB26180-B05 |
| VI | *Potamyia flavata* | Hydropsychidae (11.6)\|INDOBIOSYS-CCDB26127-G10 | *Potamyia flavata*\| INDOBIOSYS-CCDB30314-B05, n=2 |
| VII | *Chimarra* sp. | Philopotamidae (13.11)\|INDOBIOSYS-CCDB25378-F02 | *Chimarra* sp.\| INDOBIOSYS-CCDB26178-F07 |
| VIII | *Chimarra briseis* | Philopotamidae (20.6)\| INDOBIOSYS-CCDB24595-H05  Philopotamidae (20.2)\| INDOBIOSYS-CCDB26127-C01  Philopotamidae (20.15)\| INDOBIOSYS-CCDB26127-D09 | *Chimarra briseis*\| INDOBIOSYS-CCDB26178-F10  *Chimarra briseis*\| INDOBIOSYS-CCDB26178-F12  *Chimarra briseis*\| INDOBIOSYS-CCDB26178-G01  *Chimarra* sp.\| INDOBIOSYS-CCDB30314-G12 |
| IX | *Agapetus* sp. */ Glossosoma javanicum-*complex | *Agapetus* sp. (9.5)\|INDOBIOSYS-CCDB24595-D04 | *Glossosoma javanicum\|* INDOBIOSYS-CCDB26178-E06 |
|  |  | *Agapetus* sp. (9.9)\|INDOBIOSYS-CCDB24595-D05 |  |
| X | *Lepidostoma diehli/jacobson-*complex | Lepidostomatidae (15.3)\| INDOBIOSYS-CCDB24595-F01 | *Lepidostoma* sp.\| INDOBIOSYS-CCDB25378-F06 |
|  |  | Lepidostomatidae (20.9)\| INDOBIOSYS-CCDB24595-H02 | *Lepidostoma* sp.\| INDOBIOSYS-CCDB25378-F08 |
|  |  | Lepidostomatidae (15.10)\| INDOBIOSYS-CCDB25378-C04 | *Lepidostoma diehli*\| INDOBIOSYS- CCDB30314-C09 |
|  |  | Lepidostomatidae (15.5)\| INDOBIOSYS- CCDB25378-F03 | *Lepidostoma jacobsoni*\| INDOBIOSYS- CCDB30314-G11 |
| XI | *Lepidostoma sp.* | Lepidostomatidae (20.9.II)\| INDOBIOSYS-CCDB24595-H03 | *Lepidostoma sp. \|INDOBIOSYS-CCDB30314-E08* |
| XII | *Goera conclusa* | *Goera* sp. (3.4)\| INDOBIOSYS-CCDB26127-G03 | *Goera conclusa*\| INDOBIOSYS-CCDB26180-E06 |
|  |  | Goeridae (3.3.I)\| INDOBIOSYS-CCDB25378-H02 | *Goera conclusa*\|INDOBIOSYS-CCDB26180-E07 |
|  |  | Goeridae (3.3.IV)\| INDOBIOSYS-CCDB25378-H05 |  |
|  |  | Goeridae (3.3.II)\| INDOBIOSYS-CCDB25378-H03 |  |
| XIII | *Adicella* sp. | Leptoceridae (20.2)\| INDOBIOSYS-CCDB26127-H02 | *Adicella* sp.\|INDOBIOSYS-CCDB26180-A08 |
| XIV | *Oecetis tripunctata* | Leptoceridae (7.1)\| INDOBIOSYS-CCDB25378-C07 | *Oecetis tripunctata\|* INDOBIOSYS-CCDB26180-C07 |
|  |  |  | *Oecetis tripunctata*\| INDOBIOSYS-CCDB26180-E04 |
|  |  |  | *Oecetis tripunctata*\| INDOBIOSYS-CCDB26180-C05 |
|  |  |  | *Oecetis tripunctata*\| INDOBIOSYS-CCDB26180-C06 |
| XV | *Trichosetodes handschini/Setodes musagetes-*complex | Leptoceridae (6.3)\| INDOBIOSYS-CCDB25378-E03 | *Trichosetodes handschini*\| INDOBIOSYS-CCDB26179-G09 |
|  |  | Leptoceridae (6.12)\| INDOBIOSYS-CCDB25378-F10 | *Setodes musagetes*\| INDOBIOSYS-CCDB30314-D06 |
|  |  | Leptoceridae (6.3)\| INDOBIOSYS-CCDB25378-G10 | *Setodes musagetes*\| INDOBIOSYS-CCDB30314-F02 |
| XVI | *Setodes* sp. | Leptoceridae (3.3)\|INDOBIOSYS-CCDB25378-D03 | *Setodes* sp.\| INDOBIOSYS-CCDB26179-G10, n=3 |
|  |  |  | *Setodes* sp.\| INDOBIOSYS-CCDB26179-G11 |
| XVII | *Rhcophila sp.* | Rhyacophilidae (14.4.I)\|INDOBIOSYS-CCDB24595-E09 | *Rhyacophila sp.* \|INDOBIOSYS-CCDB30314-E06 |
| XVIII | *Glossosoma javanicum* | *Glossosoma* sp. (13.1)\|INDOBIOSYS-CCDB25378-G08 | *Glossosoma javanicum\|* INDOBIOSYS-CCDB26179-E06 |
