# Supplementary figures and images for: DNA barcoding unveils a high diversity of caddisflies (Trichoptera) in the Mount Halimun Salak National Park (West Java; Indonesia)
Source: PeerJ. 2022 Dec 12;10:e14182. doi: 10.7717/peerj.14182 (PMC9753737; doi:10.7717/peerj.14182)

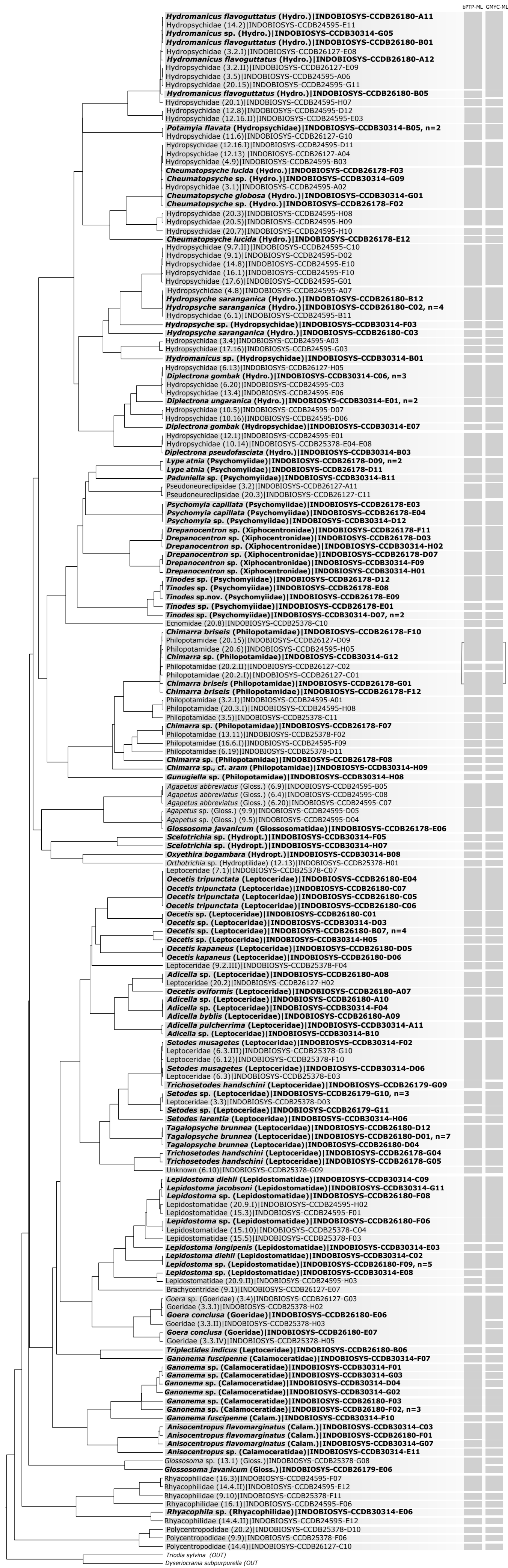

Supplement: Supplemental Information 3 — Results of GMYC and bPTP analysis are illustrated by vertical bars. N = number of haplotypes collapsed to one sequence, n= number of haplotypes collapsed to one sequence. [file peerj-10-14182-s003.pdf]
