# Supplementary material for: DNA barcoding unveils a high diversity of caddisflies (Trichoptera) in the Mount Halimun Salak National Park (West Java; Indonesia)
Source: PeerJ. 2022 Dec 12;10:e14182. doi: 10.7717/peerj.14182 (PMC9753737; doi:10.7717/peerj.14182)
Supplement: Supplemental Information 5 — The analysis was performed for (A,B) all Trichoptera sequences, (C,D) Calamoceratidae, (E,F) Leptoceridae, (G,H) Hydropsychidae, (I,J) Philopotamidae, (K,L) Lepidostomatidae, and (M,N) Psychomyiidae. [file peerj-10-14182-s005.pdf]

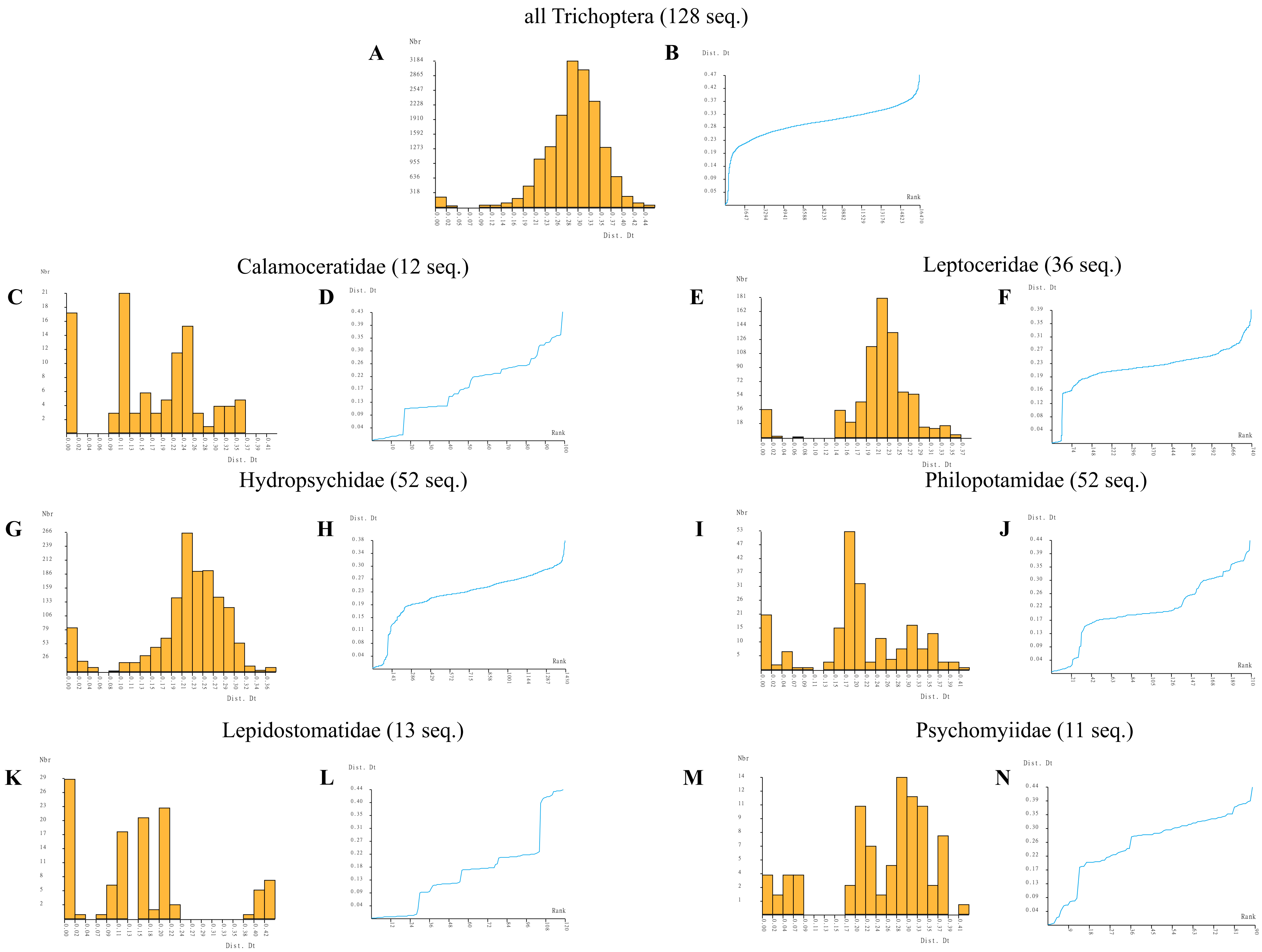
all Trichoptera (128 seq.)
A
Nbr
Dist. Dt
B
Dist. Dt
Rank
Calamoceratidae (12 seq.)
C
Nbr
Dist. Dt
D
Dist. Dt
Rank
Leptoceridae (36 seq.)
E
Nbr
Dist. Dt
F
Dist. Dt
Rank
Hydropsychidae (52 seq.)
G
Nbr
Dist. Dt
H
Dist. Dt
Rank
Philopotamidae (52 seq.)
I
Nbr
Dist. Dt
J
Dist. Dt
Rank
Lepidostomatidae (13 seq.)
K
Nbr
Dist. Dt
L
Dist. Dt
Rank
Psychomyiidae (11 seq.)
M
Nbr
Dist. Dt
N
Dist. Dt
Rank
