# Supplementary material for: DNA barcoding unveils a high diversity of caddisflies (Trichoptera) in the Mount Halimun Salak National Park (West Java; Indonesia)
Source: PeerJ. 2022 Dec 12;10:e14182. doi: 10.7717/peerj.14182 (PMC9753737; doi:10.7717/peerj.14182)
Supplement: Supplemental Information 6 — The analysis was perfomed for (A) all Trichoptera sequences, (B) Calamoceratidae, (C) Leptoceridae, (D) Hydropsychidae, (E) Philopotamidae, (F) Lepidostomatidae, and (G) Psychomyiidae. The red vertical line represents the threshold time between inter- and intraspecific branching. N = number of reconstructed lineages. Time = proportion of time from taxon origin to present. [file peerj-10-14182-s006.pdf]

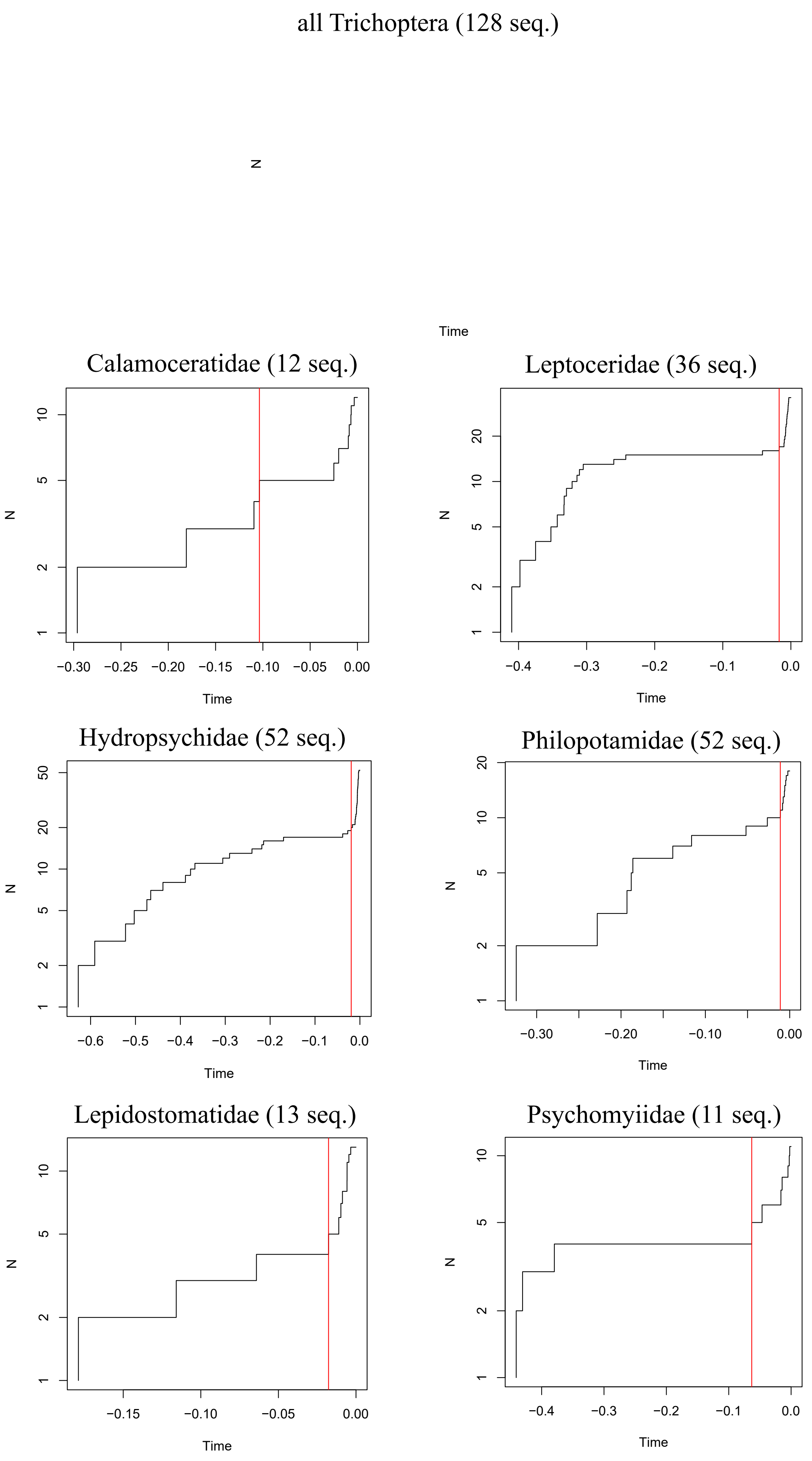

all Trichoptera (128 seq.)
N
Time
Calamoceratidae (12 seq.)
Leptoceridae (36 seq.)
Hydropsychidae (52 seq.)
Philopotamidae (52 seq.)
Lepidostomatidae (13 seq.)
Psychomyiidae (11 seq.)
N
Time
